# Gamified spatial‑participatory model for urban social cohesion in a post‑conflict city: Bridging accessibility, inclusion, and intergroup trust through co‑design in Kabul

**DOI:** 10.1371/journal.pone.0358692

**Published:** 2026-10-27

**Authors:** Aimal Formolly, Mohammad Hossain Saraei, Ebrahim Siahi

**Affiliations:** 1 Department of Geography, Yazd University, Yazd, Yazd Province, Iran; 2 Department of Urban Design, Payame Noor University, Tehran, Tehran Province, Iran; Tallinn University of Technology School of Engineering: Tallinna Tehnikaulikool Inseneriteaduskond, ESTONIA

## Abstract

Post‑conflict cities exhibit profound socio‑spatial fractures, institutional weakness, and eroded public trust. Conventional planning repeatedly fails to address ethnic segregation, accessibility disparities, and social fragmentation. The aim of this study is to propose and empirically validate an integrated model, the Gamified Spatial-Participatory Model (GSPM), that combines spatial justice theory, participatory co-design, and conflict-sensitive gamification to foster social cohesion. Using Kabul, Afghanistan, as a paradigmatic case, a sequential mixed‑methods design was employed. Quantitative spatial diagnostics mapped severe accessibility deficits, while qualitative inquiry identified deep trust deficits and cultural barriers; these jointly informed gamified co‑design workshops as the intervention. Pilot results provide strong preliminary evidence of GSPM efficacy: 90% of participants reported substantially increased engagement; gamification correlated strongly with intergroup trust (r = 0.76, p < 0.01); perceived accessibility improved by 38% in intervention areas. Critically, 70% of women and 55% of ethnic minorities reported meaningfully stronger belonging. The study demonstrates that this approach shifts planning epistemology from technocratic to deeply human, operationalising spatial justice in fragile settings, and offers transferable principles for fostering inclusion in divided urban contexts.

## 1. Introduction

Urban landscapes scarred by prolonged conflict present a profound test for contemporary planning theory and practice. These cities where the material fabric is interwoven with the legacies of violence, displacement, and institutional collapse epitomize what scholars term “urbicide”: the deliberate destruction of urbanity as a mode of social life [1,2]. In such contexts, the conventional metrics of urban dysfunction fall short; here, socio-spatial fragmentation is not merely a byproduct of market forces or poor governance but is often a deliberate strategy and a lasting consequence of war. The challenges are synergistic: ethnic enclaves become fortified, public trust evaporates, and access to essential services transforms from a municipal responsibility into a manifestation of structural violence. The ethical imperative of Sustainable Development Goal 11 to create inclusive, safe, resilient, and sustainable cities remains a distant abstraction when confronted with the complex realities of divided cities like Kabul, Mogadishu, or Sana’a.

Existing literature on post-conflict urbanism has made significant strides, yet critical gaps persist. A substantial body of work from Latin America, Africa, and Asia has meticulously documented patterns of segregation, informality, and uneven service access in cities like São Paulo, Johannesburg, and Mumbai [3–8]. These studies reveal how socio-spatial inequalities are reproduced. Meanwhile, critical urban theory, from Lefebvre’s [9] “right to the city” to Soja’s [10] “spatial justice,” provides a powerful normative framework for demanding equitable urban futures. However, a persistent disconnect remains between this rich diagnostic and theoretical scholarship and the development of actionable, culturally grounded methodologies for intervention in fragile, post-conflict settings. Much of the planning response remains siloed, oscillating between technocratic, top-down infrastructure projects and socially oriented community consultations that often fail to address deep-seated distrust or translate participation into tangible spatial equity. The literature on “domicide” the destruction of home as a psychological and social anchor further underscores that recovery is not just physical but profoundly existential [11].

This research directly confronts this critical gap. It posits that fostering social cohesion in a fractured city requires moving beyond isolated disciplinary approaches to forge an integrated, operational model. This research investigates: What are the operational mechanisms through which the Gamified Spatial-Participatory Model (GSPM) integrating spatial diagnostics, ethnographic inquiry, and conflict-sensitive gamification transforms participatory planning to simultaneously build intergroup trust and co-produce more equitable spatial outcomes in a securitized, divided city? Kabul, Afghanistan, serves as a paradigmatic and urgent case study. In the current post‑2021 governance landscape, formal municipal planning institutions remain fragmented and severely under‑resourced; community councils (shuras) and neighbourhood representatives (maliks) have assumed de facto responsibility for mediating land use, service access, and local development priorities, often in coordination with international NGOs and UN agencies. The GSPM was deliberately designed to operate within this hybrid institutional environment, providing a methodology that enables these diverse actors to move from diagnostic mapping to actionable, co‑produced spatial interventions. Decades of conflict have crystallized a stark socio-spatial order: entrenched ethnic enclaves of Tajiks, Pashtuns, and Hazaras; a poverty rate exceeding 32%; and a legacy of politicized disinvestment [12,13]. In Kabul, a journey to a clinic or school is not a simple logistical calculation but a traversal of visible and invisible boundaries checkpoints, ethnic territories, and landscapes of fear. This study bridges this ambition-action divide through the GSPM. The framework integrates three core pillars: 1) Quantitative Spatial Diagnosis, using advanced GIS analytics and security-weighted accessibility modeling to objectively map inequities; 2) Qualitative Ethnographic Inquiry, to ground spatial data in lived experience and identify cultural and psychological barriers; and 3) Conflict-Sensitive Gamified Co-Design, which transforms participatory planning into collaborative, rule-based engagement to overcome distrust and incentivize intergroup cooperation. By synthesizing spatial demography, community psychology, and participatory planning, the GSPM shifts the paradigm from siloed analysis or consultation to a deeply human-centered, interdisciplinary process of co-creation.

The significance of this integrated design is threefold, moving from theoretical advancement to practical proof. First, it advances theoretical discourse by operationalizing concepts like spatial justice and the right to the city in one of the world’s most challenging urban environments, moving them from critique to applied methodology. Second, it provides a replicable, evidence-based model the GSPM itself for urban practitioners and policymakers in post-conflict and deeply divided cities worldwide. Finally, its third pillar demonstrates the transformative mechanism: culturally embedded, gamified participation achieves levels of inclusion and trust-building that traditional methods consistently fail to reach, particularly among women and ethnic minorities. In doing so, this study argues that rebuilding cohesive urban futures in the shadow of conflict demands not just technical solutions but a fundamental reimagining of the planning process itself as an integrative, equitable, and empowering practice. The resulting proof‑of‑concept, developed and tested through fieldwork (2024–2025) under Kabul’s post‑2021 governance constraints, is explicitly intended to equip municipal authorities, community councils, and humanitarian agencies with a replicable, evidence‑based model for inclusive forward planning.

## 2. Literature review

The challenge of fostering social cohesion and equitable accessibility in fragmented cities sits at the intersection of multiple scholarly discourses. A rigorous examination of existing literature reveals three predominant, yet often disconnected, streams: critical theories of post-conflict urbanism and spatial violence; empirical analyses of socio-spatial segregation and informality across the Global South; and evolving methodologies for participatory planning and community engagement. This review synthesizes these bodies of work to construct a nuanced theoretical and practical landscape. It argues that while these literatures offer powerful diagnostics and normative frameworks, a significant gap persists in actionable, interdisciplinary models that can translate critique into constructive, culturally-grounded intervention in the unique crucible of the post-conflict city. This gap is where the present study, through its GSPM, positions its central contribution.

### 2.1. Discussion of concepts

#### 2.1.1. Urbanism in the shadow of conflict: From diagnosis to structural violence.

The first pillar engages literature viewing cities as targets and products of conflict. Beyond generic fragility, urbicide the deliberate killing of the city as a pluralistic, democratic entity frames destruction of mosques, markets, and public spaces as an assault on social fabric [2,14]. Domicide complements this by highlighting systematic home destruction to inflict psychological trauma and unravel community bonds [11]. In Kabul, conflict-induced displacement and politicized reconstruction aid produce a slow domicide. Critical geography further shows warfare’s spatial embedding: Graham’s [15] “bulldozer and the bomb” merges urban planning with military strategy, creating permanent landscapes of control and exclusion. Parks [16] conceptualizes vertical power surveillance, aerial bombardment, fortified enclaves reshaping lived experience. Thus, Kabul’s checkpoints and ethnic enclaves are politicized spatiality where identity and historical power mediate access. The 24 km travel burden from District 22 exemplifies structural violence, where political structures cause measurable harm by preventing marginalized groups from meeting basic needs.

#### 2.1.2. Segregation, informality, and access: A global south perspective.

The second pillar situates Kabul within Global South urban inequality, decentering Western views. Research from Latin America [5,17] demonstrates how fear and inequality fragment cities. South Africa’s apartheid legacy engineered extreme spatial injustice, with townships distanced from economic centers, persisting post-1994 [6,7]. In India, Roy [8] theorizes informality not as anomaly but as dominant urban production, where land and service access is negotiated through patronage and ethnicity. These perspectives show that Kabul’s ethnic enclaves and service deserts are extreme expressions of widespread phenomena, not unique. Local studies [12,18] confirm patterns resonant with Johannesburg and São Paulo, placing Kabul within a continuum of urban marginalization intensified by decades of warfare.

#### 2.1.3. The promise and peril of participatory planning.

The third stream examines participatory planning. Normative theories like Lefebvre’s [9] “right to the city” and Soja’s [10] “spatial justice” advocate inclusive, democratic urbanism against technocracy. Yet critical literature documents frequent failure in divided societies: participation can become tokenistic “tyranny” [19], co-opted by elites or NGOs, reinforcing inequalities. In contexts of ethnic distrust and weak institutions, conventional meetings fail to engage marginalized groups, especially women and minorities [20], because they cannot overcome social-psychological barriers of fear, distrust, and historical grievance as formidable as physical ones. Gamification, the integration of game-design elements such as points, rules, and rewards into non-game processes, and serious games, fully fledged games designed for purposes beyond entertainment, offer an alternative by lowering tension, incentivizing cooperation, and creating safe spaces through structured roles and challenges [21]. Within urban planning, serious games have emerged as participatory tools for scenario-building, stakeholder negotiation, and collaborative spatial design, yet their application in post-conflict contexts remains largely unexplored. Studies in Brazil and India show enhanced youth engagement [22], but most applications remain in stable Western contexts, lacking testing in high-stakes post-conflict environments where violence legacies constrain interaction. In urban planning, this distinction is operational: gamification typically overlays incentive structures (points, badges, leaderboards) onto existing consultation formats to boost attendance or compliance, whereas serious games constitute self-contained, rule-bound simulations that restructure the decision-making process itself. For example, a gamified workshop might award points for contributing ideas to a conventional charrette, while a serious game replaces the charrette entirely with a role-play scenario in which participants negotiate land-use trade-offs under explicit rules and win conditions. In post-conflict settings, serious games are particularly valuable because they create bounded, low-risk environments where historically opposed groups can practice cooperation, test scenarios, and co-produce spatial solutions without the immediate consequences of real-world negotiation.

### 2.2. Discussion of relationships and identification of gaps

The relationship among these literatures is one of parallel existence. Urbicide/structural violence theories diagnose the problem; Global South segregation studies provide measurement analogues and methodologies; critical participatory planning highlights procedural shortcomings. No cohesive framework integrates conflict urbanism’s diagnostic depth and spatial inequality’s analytical rigor with an innovative, conflict-sensitive participatory methodology a holistic gap. Lefebvre and Soja inspire the ‘what’ but not the ‘how’ in a landscape of domicide; segregation studies map inequality but stop at the threshold of intervention; gamification proposes engaging methods but lacks grounding in post-conflict spatial and psychological realities. Rebuilding social cohesion in Kabul thus requires transcending silos: a model that is spatially literate, ethnographically grounded, and procedurally innovative. Current scholarship offers pieces but not an assembled, actionable post-conflict framework.

Therefore, this study contributes the GSPM, empirically validated. Synthesizing these literatures, it employs spatial analysis tools from Global South urban studies, is informed by structural violence and spatial justice, and advances participatory planning through conflict-sensitive gamification to bridge trust gaps and enable genuine co-design, moving from diagnosis to repair. The GSPM, depicted in Fig 1, integrates spatial diagnostics, ethnographic inquiry, and conflict-sensitive gamified co-design into a unified framework wherein quantitative mapping of inequities, lived-experience narratives, and rule-based collaborative games jointly foster intergroup trust and equitable spatial outcomes. This approach also distinguishes itself from technical advances: while the Spatial Proximity Chain (SPC) method quantifies segregation via built barriers, the security-weighted 2SFCA framework captures dynamic, conflict-based impedance; human-in-the-loop platforms like InclusiViz demonstrate interactive analytics for segregation mitigation, extensible to future GSPM iterations; and compared to serious-game frameworks like EquiCity, GSPM innovates by embedding conflict-sensitive facilitation and identity-aware role-play for high-stakes, low-trust environments, beyond classroom prototyping. To make the literature scope and selection criteria explicit, Table 1 synthesises the key thematic clusters, representative authors, geographic focus (with deliberate emphasis on the Global South), and temporal range of the reviewed works. The accompanying narrative outlines the search strategy employed.

**Table 1:** Summary of key literature, themes, and geographic scope.

Thematic Cluster	Key Writers	Geographic Focus/ Setting	Key Dates	Contribution to This Study
Post-conflict urbanism, urbicide, domicide, and structural violence	[1,2,11,14–16]	Global/ theoretical; Iraq, former Yugoslavia, Kabul	2001–2025	Provides the diagnostic language of spatial violence and the deliberate destruction of urban fabric, framing Kabul’s fractures as structural rather than incidental.
Socio-spatial segregation, informality, and unequal access in the Global South	[4–8,12,17,18]	São Paulo, Buenos Aires, Johannesburg, Mumbai, Indian neighbourhoods, Kabul	2003–2025	Supplies comparative empirical evidence on segregation and service inequities; positions Kabul within a continuum of Global South marginalisation rather than as an isolated case.
Normative theories of spatial justice and the right to the city	[9,10]	Primarily Western/ theoretical	1991, 2010	Establishes the ethical imperative for equitable urban processes but lacks procedural guidance for post-conflict settings; serves as the normative anchor of the GSPM.
Participatory planning critique (tyranny, exclusion)	[19,20,23]	Western, Ecuador	1969–2023	Highlights the recurrent failures of conventional participation tokenism, elite capture, exclusion of women and minorities and the psychological barriers that gamification must overcome.
Gamification and serious games in participatory contexts	[21,22]	Latin America, India, Brazil; conceptual	2024–2025	Demonstrates the potential of game-based methods to boost engagement and collaboration, but applications remain concentrated in stable environments and require adaptation for post-conflict realities.

**Fig 1:**
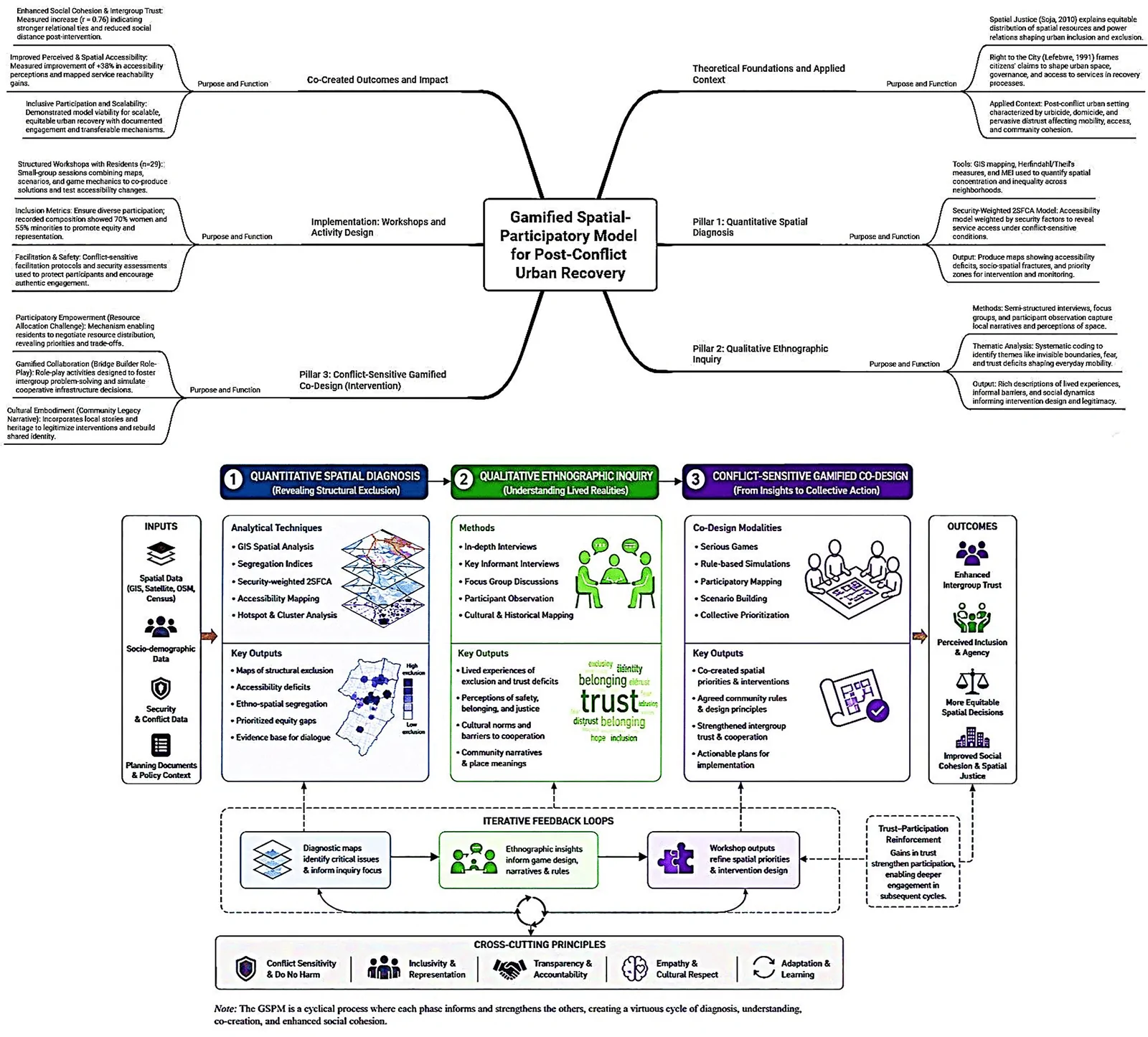
a. The normative purpose and intended outcomes of the GSPM. b. The methodological sequence and feedback mechanisms of the GSPM. Source: Authors conceptualization and design.

#### 2.2.1. Literature search strategy.

Relevant literature was identified through systematic searches of Scopus, Web of Science, and Google Scholar, using Boolean combinations of the following keyword groups: (“post-conflict urban” OR “urbicide” OR “domicide” OR “spatial justice” OR “fragile city”) AND (“segregation” OR “accessibility” OR “informality”) AND (“Global South” OR “Kabul” OR “Afghanistan” OR “divided city”); (“participatory planning” OR “co-design” OR “gamification” OR “serious games”) AND (“urban” OR “spatial”) AND (“conflict” OR “post-conflict” OR “fragile”). Snowball sampling of reference lists extended the corpus. Inclusion prioritised works addressing spatial inequality, participatory methods, or game-based engagement in the Global South or fragile contexts, with no date restriction, yielding the core literature summarised below.

### 2.3. Conceptual framework: The GSPM – bridging justice, diagnosis, and co-creation

The model’s normative purpose and its methodological architecture are separated into two complementary diagrams for clarity (Fig 1a and 1b). Fig 1a presents the overarching purpose of the GSPM: to move from a condition of fractured urban space and eroded trust manifested in urbicide, ethnic segregation, and stark accessibility deficits toward enhanced social cohesion and spatial equity, mediated by collaborative co‑design. The fig positions spatial justice [10] and the right to the city [9] as the ethical foundation, and identifies the key outcome domains: improved intergroup trust, perceived inclusion and agency, and more equitable spatial decisions. Fig 1b depicts the methodological workflow that operationalises this purpose. It sequences three integrated pillars: (1) Quantitative Spatial Diagnosis (GIS, segregation indices, security‑weighted 2SFCA) to map structural exclusion; (2) Qualitative Ethnographic Inquiry (in‑depth interviews, key informant interviews, focus groups) to surface lived experiences of trust deficits and cultural barriers; and (3) Conflict‑Sensitive Gamified Co‑Design workshops, where diagnostic insights are translated into serious games and rule‑based collaborative exercises. The diagram highlights iterative feedback loops: ethnographic findings inform game design, workshop outputs refine spatial priorities, and gains in trust reinforce participation in subsequent cycles.

The model provides a procedural pathway from critique to co-creation. It posits that quantitative spatial analytics render visible the material structures of exclusion, while ethnographic engagement elucidates the subjective realities of “invisible boundaries” and eroded trust. This dual diagnostic foundation is not an end in itself but a critical input to calibrate the final, transformative phase: Conflict-Sensitive Gamified Co-Design. This phase is theorized as a structured social forum that re-engineers the participatory encounter. By transmuting analytical insights into rule-based rituals for collective imagination, it aims to overcome the “tyranny of participation” and incentivize cooperation across deep-seated divides. The sequential flow (Diagnosis → Empathetic Understanding → Targeted Co-Creation) is iterative, designed to create a virtuous cycle where improved social trust and spatial equity reinforce one another. Thus, the GSPM (see Fig 1) reframes social cohesion not as a vague outcome but as a spatially mediated practice, offering a replicable blueprint for diagnosing and repairing urban fracture.

## 3. Methods

Kabul was selected as a critical case [24] for testing the GSPM because it concentrates the very conditions that the model is designed to address: entrenched ethnic segregation, extreme accessibility disparities, severely eroded public trust, and a planning landscape fragmented between informal community authority and weakened formal institutions. The city’s recent history of continuous conflict, its sharp socio-spatial polarisation, and the near-total absence of effective participatory mechanisms make it an extreme, paradigmatic instance of post-conflict urban fracture. If the GSPM can demonstrate efficacy in this highly constrained, securitised environment, then its transferability to less extreme but similarly divided cities becomes analytically plausible. This logic follows the case-selection strategy of identifying a “most-difficult” setting wherein the mechanisms of interest are starkly visible [25].

### 3.1. Research context, logistics, and ethics

Conducted from 15 January 2024 to 22 September 2025 under the Islamic Emirate of Afghanistan, all fieldwork was facilitated by local shuras. Workshops were co‑facilitated by five trained local advocates (3 male, 2 female) ensuring cultural protocols. All interviews and focus groups were conducted in Dari or Pashto (Afghanistan national languages), depending on participant preference, by the lead researcher and the trained facilitators. In‑depth interviews (IDIs) lasted between 45 and 90 minutes; key informant interviews (KIIs) ranged from 30 to 60 minutes; focus group discussions (FGDs) lasted 90–120 minutes. With separate verbal consent for audio-recording, IDIs and FGDs were digitally recorded using encrypted devices, transcribed verbatim in the original language, and then translated into English by a bilingual research assistant. Back‑translation checks were performed on 20% of transcripts to ensure accuracy. KIIs were documented through detailed contemporaneous notes rather than recordings, as many officials expressed discomfort with being audio‑recorded. The gamified workshops were audio‑recorded for group interactions, and facilitators compiled structured observation logs immediately after each session. Practical challenges included intermittent electricity, security-related movement restrictions, and the need to reschedule several sessions due to community events; these constraints shaped a flexible, adaptive fieldwork rhythm. The study area, indicating the 22 districts, the Chendawel pilot neighbourhood, and the control area, is provided as Fig 2 for spatial orientation. Verbal informed consent, witnessed by a same‑gender facilitator, was obtained without recording identifying details; gender‑segregated workshops were synthesised by facilitators. Key Informant Interviews (n = 40) included former municipal planners (pre‑2021), current de facto community‑level officials (maliks), and NGO staff.

**Fig 2:**
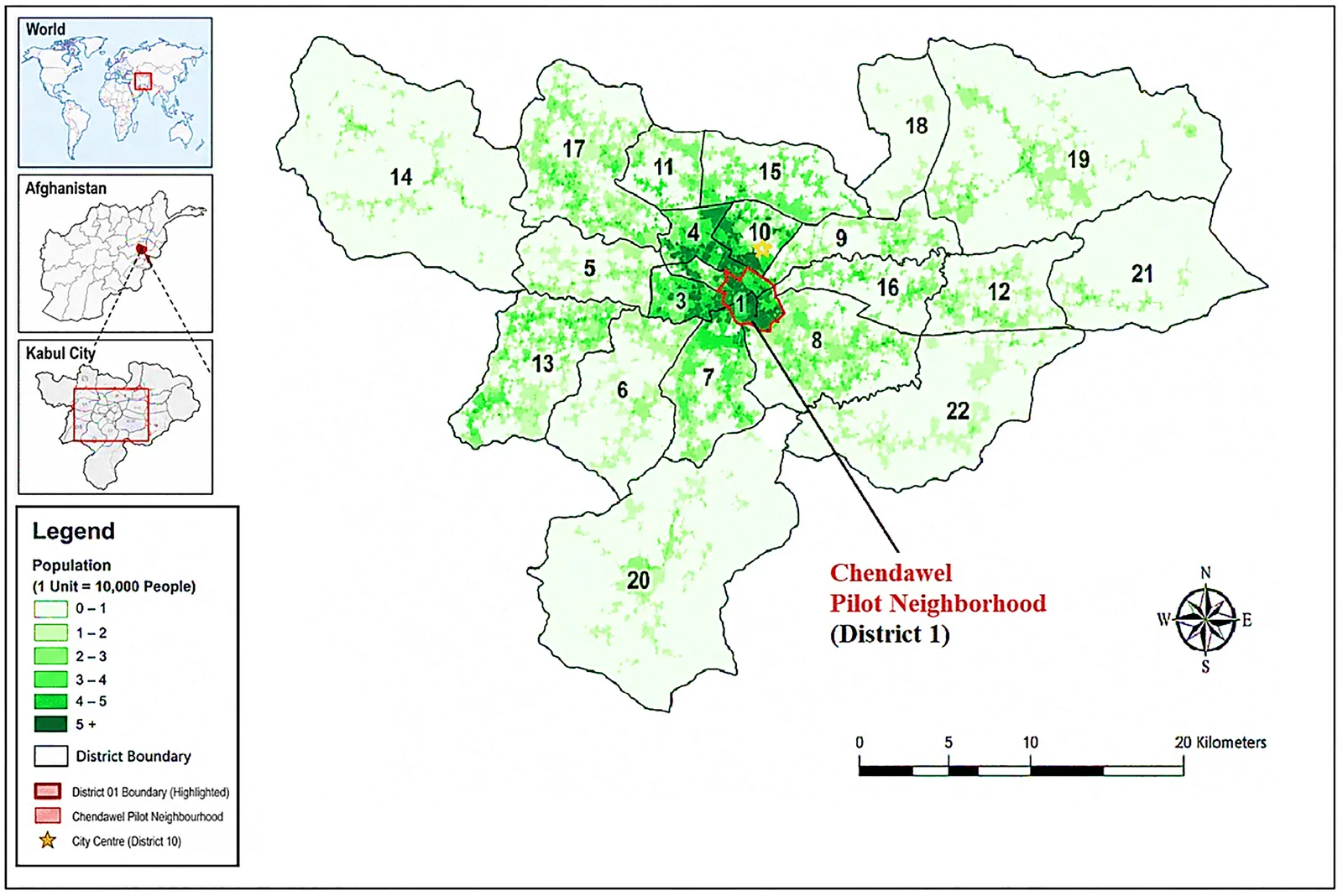
Spatial distribution of population density across the Kabul metropolitan area, with the Chendawel pilot neighbourhood (red outline) in District 10 (historic city centre) marked for spatial reference. Map created by the authors using ArcGIS 10.4.1. Basemap features are derived from OpenStreetMap® data (© OpenStreetMap contributors, ODbL; see https://www.openstreetmap.org/copyright). Terrain relief is based on SRTM data (public domain, NASA/USGS). Demographic data are from the authors’ own dataset. This figure is original work by the authors and is published under the Creative Commons Attribution 4.0 International (CC BY 4.0) license.

### 3.2. Theoretical framework and analytical approach

An integrated framework synthesising spatial justice [10], the right to the city [9], and conflict‑sensitive participatory design was operationalised as the GSPM, hypothesising that this integrated approach tangibly improves accessibility and cohesion. A sequential explanatory mixed‑methods design progressed from quantitative spatial diagnosis and qualitative inquiry to participatory intervention design and evaluation, securing methodological triangulation.

### 3.3. Data inventory and reproducibility

Transparency is assured by a complete data ledger (Table 2). Spatial analyses employed ArcGIS Pro 3.0 and QGIS 3.28; statistics used IBM SPSS Statistics 27 and the PROCESS macro.

**Table 2:** Data inventory and sources.

Data Category	Specific Dataset/Variable	Producer/ Source	Year	Unit of Analysis	Coverage
Demographic & Socioeconomic	Total Population, Age-Sex Structure	Nat. Statistics and Info. Authority (NSIA) of Afghanistan [26]	2024 (Est.)	District (N = 22), Neighborhood	Kabul Province
	Ethnicity Distribution	CSO & UN-Habitat (2017) [27]; Validated via Satellite Settlement Pattern Analysis (see 3.3)	2017 (base), 2025 (validated).	Neighborhood	Kabul City
	Poverty Rate, Income Proxy	World Bank (2024) [13]; NSIA SWIFT-3 Survey	2024	District, Household	Kabul Province
Spatial & Infrastructure	Admin Boundaries, Road Network	Kabul Municipality; OpenStreetMap	2024	Polygon, Line	Kabul City
	Service Points: Health, Schools, Parks	Ministries of Public Health, Education; Kabul Municipality	2022 (base), 2024 (validated).	Point	Kabul City
	Land Use/ Land Cover (LULC)	Sentinel-2 Satellite Imagery (10m)	2025	Raster	Kabul Region
Conflict & Security	Conflict Event Locations	Armed Conflict Location & Event Data Project (ACLED) [28]	2024-2025	Point	Afghanistan
	Security Checkpoint Locations	Field Mapping & Key Informant Interviews	2024	Point	Pilot Area
Primary Qualitative & Survey	In-depth Interview (IDI) Transcripts	Researcher-conducted	2024-2025	Individual	5 Districts -Purposive sample (n = 14 residents).
	Key Informant Interview (KII) Notes	Researcher-conducted	2024	Individual	Kabul City -Purposive sample (n = 40).
	Focus Group Discussion (FGD) Transcripts	Researcher-facilitated	2025	Group	Chendawel- Three FGDs (n = 29 total).
	Pre-/Post-Intervention Survey	Researcher-administered	2024 (Pre), 2025 (Post)	Household	Chendawel (Pilot), Control Area- Stratified random sample (n = 137 per area; total N = 274, 98% response).

### 3.4. Instrument reliability and validity

Internal consistency was assessed using Cronbach’s alpha on the full pre‑intervention sample (N = 274). The perceived accessibility composite (5 items on service reachability, safety, travel time, affordability, and dignity) yielded α = 0.84, and the social cohesion scale (8 items adapted from Putnam’s social capital index, covering trust, reciprocity, and collective efficacy) yielded α = 0.79, indicating good reliability [29]. Additionally, the intergroup trust subscale (3 items assessing willingness to cooperate with, share resources with, and feel safe among members of other ethnic groups) demonstrated acceptable reliability (α = 0.76). Construct validity is supported by significant negative correlations with the neighbourhood segregation index (MEI) and positive correlations with the wealth index, consistent with theoretical expectations. Face validity was established through expert review by the local co‑facilitation team and pilot testing with 12 residents, whose feedback informed wording refinements. For the trust measure specifically, convergent validity was supported by a strong correlation with a single-item measure of generalised trust (r = 0.61, p < 0.001) administered in the same survey.

### 3.5. Derivation and validation of key variables

In the absence of an official census, ethnicity serves as a spatial heuristic. The 2016 Kabul City Profile was updated via satellite settlement‑morphology analysis (Maxar high‑resolution imagery) cross‑referenced with (a) 40 KIIs confirming local ethnic majorities, (b) geolocated ethnic‑affiliated cultural sites, and (c) ground‑truthing from 14 IDIs [27]. Discrepancies were conservatively resolved favouring KII evidence; data were aggregated to the neighbourhood level to mitigate ecological fallacy, with shura ethical approval. A wealth index was constructed by PCA on housing material quality (satellite classification), access to piped water/electricity, and asset ownership. The security‑weighted 2SFCA modified the standard method via impedance layers: a 50% speed reduction within 1 km of ACLED conflict events and a + 15‑minute checkpoint delay, parameters derived from 15 KIIs with drivers, NGO logisticians, and community leaders [28]. Sensitivity analysis (conflict buffer 0.5–1.5 km, checkpoint delay +10–20 min) showed city‑wide mean accessibility varying <±5% (S1 Table); GPS validation was infeasible for security reasons.

The ethnicity variable was derived through a four‑step triangulation procedure. First, initial ethnic clusters were drawn from the 2016 Kabul City Profile [27]. Second, high‑resolution satellite imagery (Maxar) was used to classify settlement morphology, distinguishing, for instance, the courtyard‑based compounds characteristic of traditional Pashtun layouts from the denser, inward‑facing patterns of Hazara neighbourhoods. Third, these patterns were cross‑validated with 40 key informant interviews that confirmed majority ethnicity per neighbourhood. Finally, ground‑truthing was conducted during 14 in‑depth interviews. All data were aggregated to the neighbourhood level to prevent any identification of individuals or households. This approach does not infer any individual’s ethnicity; it estimates neighbourhood‑level composition as a spatial heuristic for understanding structural inequality, a practice endorsed by humanitarian mapping guidelines when census data are unavailable or politicised. It must be explicitly acknowledged that this neighbourhood‑level estimation procedure is subject to potential stereotyping and misclassification, and its validity depends on the convergence of the four triangulated sources rather than any single indicator. No independent, gold‑standard validation of these neighbourhood‑level ethnicity estimates was feasible given the absence of a reliable census or population register; consequently, the estimates must be interpreted as provisional and subject to revision, and this absence of formal validation is acknowledged as a methodological limitation. The procedure was reviewed and approved by the community shuras, who examined the resulting maps and confirmed that publication would not expose minority households to risk. Critically, no individual‑level ethnicity was ever collected, inferred, or stored; ethnicity appears only as a neighbourhood‑level contextual covariate in aggregate models, precluding any possibility of re‑identification.

### 3.6. Pilot and control area selection

Chendawel was purposively selected as a paradigmatic Hazara–Qizilbash enclave epitomising city‑wide socio‑spatial pathologies (historically marginalised, clear “invisible boundaries”). A demographically comparable control area was matched on dominant ethnicity (Hazara), wealth index (±0.15 SD), baseline perceived accessibility (±0.05), and security profile (all Standardized Mean Differences <0.1; S2 Table) and was located in a separate district to prevent contamination.

### 3.7. Analytical techniques: A four-pillar sequential design

#### 3.7.1. Pillar 1: Quantitative spatial diagnosis.

Segregation patterns were first examined through segregation analysis, in which both the Herfindahl Index (HI) and Theil’s Multigroup Entropy Index (MEI) were computed at the district and neighborhood scales using validated ethnic composition data. To capture spatial disparities in urban services, a security-weighted 2SFCA method was applied to generate neighborhood-level accessibility scores expressed as supply-to-demand ratios for healthcare, education, and park facilities. Following these measurements, statistical modeling was conducted. Initial exploration employed bivariate Pearson correlations to assess the direction and strength of associations among variables. To empirically test the core hypotheses, a multivariate ordinary least squares (OLS) regression model was then specified, incorporating the segregation indices and accessibility metrics as key predictors.


$$\begin{array}{c} HI=\text{1}-\sum_{i=\text{1}}^{n}S_{i^{\text{2}}} \end{array}$$ (1)

Here, S_*i*_ denotes the proportion of ethnic group *i* within a population comprising *n* distinct ethnic groups. The resulting index can be interpreted as the probability that two randomly selected individuals from the same area belong to different ethnic groups. Higher HI values therefore signify greater ethnic heterogeneity. To quantify the degree of ethnic segregation at the neighbourhood scale, the Multi-Ethnic Index (MEI) was employed. At the district level the primary analytical unit the MEI is computed by comparing each district with its constituent sub-district units (Output Areas, OAs), which in this study correspond to the approximately 1,053 officially recognized community-development zones and informal-settlement clusters.


$$\begin{array}{c} MEI=\;\sum_{j=\text{1}}^{m}\left[\frac{t_{j}}{T}.\;\frac{\left(E-e_{j}\right)}{E}\right] \end{array}$$ (2)

Where *E* denotes the entropy score of the district, *e*_*j*_ represents the entropy scores of the constituent neighborhoods (OAs), t_j_ is the population of OA *j*, and *T* is the total population of the district. Following Iceland [30], when the proportion of a specific ethnic group in an OA is zero, the logarithmic term is defined as zero. This convention ensures that the absence of a particular group does not artificially inflate the overall segregation measure. The MEI thus ranges from 0 to 1, with higher values reflecting greater segregation, corresponding to larger differences in ethnic composition across OAs.


$$\begin{array}{cc} Accessibilit{y\;Score}_{\;i}=\beta \text{0}\;+\;\beta \text{1}\;({Segregation\;Index}_{i})+\;\beta \text{2}\;\left(Wealt{h\;Index}_{i}\right) & \\ \;+\;\beta \text{3}\;\left(Historica{l\;Displacement}_{i}\right)+\;\beta \text{4}\;\left(Distanc{e\;to\;Center}_{i}\right)+\;\varepsilon_{i} \end{array}$$ (3)

The mediation effect of wealth on the segregation-access relationship was tested using Hayes’ PROCESS macro (Model 4) [31].

#### 3.7.2. Pillar 2: Qualitative ethnographic inquiry.

Purposive sampling selected 14 in‑depth interview (IDI) residents from six districts spanning the full segregation–accessibility range, ensuring ethnic and gender diversity, plus 40 KIIs (urban planners, officials, NGO practitioners) and three focus group discussions (FGDs, n = 29) in pilot and control areas. Semi‑structured guides explored access experiences, intergroup trust, and structural barriers until thematic saturation. Thematic analysis in NVivo 12 followed Braun & Clarke’s [32] hybrid inductive–deductive approach (inter‑coder reliability κ = 0.84).

#### 3.7.3. Pillar 3: Gamified Participatory Intervention (GSPM Application).

Diagnostic findings from Pillars 1 and 2 directly informed three serious‑game workshops in the Chendawel neighbourhood (34.5117°N, 69.1753°E), a historically marginalised Hazara–Qizilbash (Shi’a Muslim) enclave whose well‑defined community structure, cultural cohesion (including landmarks like the public Takyeh), and entrenched invisible boundaries offered a stringent test of the GSPM. Workshops were held on three consecutive Fridays in April 2025, each lasting approximately four hours, and were hosted in a community hall made available by the local shura. Participants were purposively recruited to ensure diversity in ethnicity, gender, age, and length of residence; a total of 29 residents took part across the three sessions, with 12–14 participants per workshop to maintain manageability. The sessions were co‑facilitated by the trained local advocates, who explained rules in Dari/Pashto, modelled inclusive behaviours, and managed group dynamics. Each workshop was structured around three game‑based activities, directly translating diagnostic insights into collaborative mechanics: (1) Participatory Empowerment via the “Resource Allocation Challenge.” Teams were given a large printed map of their neighbourhood overlaid with accessibility deficit scores derived from the security‑weighted 2SFCA analysis, and a limited point budget corresponding to realistic municipal investment constraints. Teams negotiated which public space to co‑design, directly confronting the spatial inequities revealed in Pillar 1. (2) Gamified Collaboration through the “Bridge Builder” role‑play. Participants were randomly assigned cross‑cutting identity profiles (e.g., a Hazara elder, a young Pashtun mother, a Tajik trader) designed to reflect the real social cleavages identified in the qualitative inquiry. The game’s win conditions required negotiating a shared design for a multi‑ethnic public facility, with points awarded for compromises that benefited the collective rather than individual roles. To ensure replicability, the operational mechanics of the Bridge Builder role‑play are detailed here. The game was played over three rounds, each lasting 40 minutes. At the outset, each participant drew a sealed role card describing a fictional but realistic character whose demographic attributes, personal history, and spatial priorities were directly derived from the qualitative themes identified in Pillar 2 (e.g., fear of crossing ethnic boundaries, gendered spatial exclusion, frustration with service deficits). Table 3 provides four illustrative role cards.

**Table 3:** Illustrative role cards for the Bridge Builder role‑play.

Role	Demographic Profile	Key Needs/ Fears	Spatial Priority
A	Hazara elder, male, long-term resident of Chendawel	Remembers violence in 1990s; distrusts outsiders; wants a safe community space exclusively for his community	A walled garden with separate entrance
B	Young Pashtun mother, recently displaced from District 22	Fears harassment when crossing into Hazara-majority areas; needs a clinic for her children	A health post with female-only hours
C	Tajik trader, male, commutes from District 2	Worried about economic marginalisation; wants market stalls accessible to all ethnic groups	A shared marketplace with parking
D	Qizilbash youth, female, student	Desires a library and a space where young women can gather without stigma	A women-friendly community library

Each round began with a spatial scenario: a printed map of a vacant plot at the boundary between two ethnic enclaves. The group was instructed to co‑design a facility on that plot using a fixed point budget (100 points), with different design features costing different amounts (e.g., a shared prayer room = 20 points, separate entrances for men and women = 15 points, a neutral‑zone café = 25 points). Points were earned individually during negotiation: 5 points for acknowledging another role’s concern, 10 points for proposing a compromise that sacrificed one’s own initial priority, and a 20‑point collective bonus if the final design included elements that bridged ethnic groups (e.g., a shared courtyard or a rotating market day for different communities). The facilitator publicly displayed a running scoreboard, which converted the negotiation into a transparent, low‑stakes competition for the highest individual and team scores. All designs had to be approved by a majority vote, preventing domination by any single role. The following abbreviated exchange, drawn from Workshop 2 and translated from Dari, illustrates how the mechanics operated:

Role A (Hazara elder): “I need a garden where our elders can sit without fear. The other community has its own places. Why must we share everything?” Role B (Pashtun mother): “If I bring my child here, I must know the entrance is safe for me. If there is a separate women’s door, I can come. Maybe we can share a clinic if the building has two sides.” Role C (Tajik trader): “I have an idea. What if we put the clinic in the middle, with two entrances one for your community, one for hers—and a small shop I can run that sells to both? I will give a discount to elders. That way I earn, she gets health, and you have your safe garden behind.” Facilitator: “Role C, you just proposed a compromise that gives up your exclusive shop space to serve both groups. That earns 10 points. The design now has a shared clinic with dual entrances and a commercial kiosk. Points to Role C.” This exchange exemplifies the rule structure: a competitive incentive (individual points) was harnessed to produce cooperative spatial outcomes, while the facilitator’s real‑time scoring made compromise tangible and rewarded. All workshop negotiations were audio‑recorded, transcribed, and coded for instances of perspective‑taking, compromise offers, and expressions of trust, forming the basis for the within‑workshop analysis reported in Section 4.3. (3) Cultural Embodiment in the “Community Legacy” narrative game. Drawing on local Islamic traditions of waqf (endowment) and shura (consultation), teams designed a community asset that would serve future generations. Non‑monetary rewards, public certificates of recognition signed by the shura elders and a communal closing ceremony were used to align incentives with cultural values of social contribution and honour. All game parameters budgets, sites, identity roles, conflict scenarios were populated with real data and tensions surfaced in the prior diagnostic phases. Facilitators documented decision processes through structured observation templates, and the plenary debrief at each workshop’s end captured participant reflections on trust, cooperation, and fairness. Audio recordings of group negotiations were transcribed and thematically analysed alongside the observation logs, providing a rich qualitative account of how game mechanics mediated intergroup interaction.

#### 3.7.4. Pillar 4: Longitudinal impact evaluation.

A stratified random sample of 137 households was surveyed in the Chendawel pilot area and 137 in a demographically matched control area (total N = 274; 98% response rate). The pre- and post-intervention surveys were administered to the same households, constituting a true panel. Attrition between waves was zero for the pilot area and three households (2.2%) for the control area; analysis used complete-case data, as the negligible attrition was deemed unlikely to introduce meaningful bias. Clustering of households by neighbourhood block was accounted for by including block-level random intercepts in sensitivity models; these did not materially alter point estimates. Difference‑in‑differences (DiD) estimation was employed to isolate the intervention effect: The DiD model was specified as follows:


$$\begin{array}{c} Y_{it}=\beta_{\text{0}}+\beta_{\text{1}}\left({Treatment}_{i}\right)+\beta_{\text{2}}\left({Time}_{t}\right)+\beta_{\text{3}}\left({Treatment}_{i}\;\times \;{Time}_{t}\right)+\;\beta_{\text{4}}{(X}_{it})+\epsilon_{it} \end{array}$$ (4)

Where Y_it_ is the outcome for household *i* at time *t*, Treatment_i_ is a binary indicator for pilot-area residence, Time_t_ is a binary indicator for the post-intervention period, and X_it_ is a vector of household-level covariates (wealth index, household size, and duration of residence) included to improve precision. Changes in pilot‑area outcomes from pre‑ to post‑intervention were compared with contemporaneous changes in the control area, with effect sizes reported as Cohen’s d and 95% confidence intervals. Paired‑sample t‑tests assessed pre‑post changes within the pilot area; independent‑sample t‑tests on change scores tested the net DiD effect, and Cohen’s d was calculated using the pooled standard deviation derived from the standard error of the net effect. No observations were missing for the primary outcome variables, and all models were checked for influential outliers using standardised residual diagnostics.

### 3.8. Ethical approval statement

The protocol received full ethical review and approval from the community councils (shuras) of the participating districts, the sole functioning ethical authority in the study context. No independent institutional review board currently operates in Afghanistan; therefore, this community-level approval constitutes the fullest ethically recognised review possible under local conditions and is explicitly acknowledged as a study limitation. The shura review covered research objectives, data collection instruments, informed consent procedures, risk assessment, and community benefit. Written minutes of approval (in Pashto/Dari) are available on request. This procedure is consistent with the Declaration of Helsinki’s provision for research in settings lacking formal IRBs, where community‑level review constitutes the best available protection and is reported here as an inherent constraint of the research environment rather than a substitute for institutional oversight. Verbal consent was used because of low adult literacy (~43%), fragile security (risks of carrying signed identifiers), cultural norms favouring witnessed verbal agreements, and the absence of a written‑consent legal framework under the de facto authorities. Verbal consent was witnessed by a same‑gender facilitator, recorded in a confidential log (participant code, date, facilitator signature, checkbox; no names), and audio‑recorded for group settings with separate permission (recordings destroyed after transcription). All procedures adhered to the Declaration of Helsinki; participants received detailed information on study aims, data handling, and the right to withdraw without consequence before providing verbal consent. The absence of a formal institutional review board is further documented in the Limitations section as a structural constraint on independent oversight in the study context.

### 3.9. Case background

#### 3.9.1. Analytical introduction: A city shaped by conflict and segregation.

Kabul exemplifies a post-conflict city where socio-spatial fragmentation is both a legacy of violence and an active exclusion mechanism. Constrained within the Hindu Kush valley, decades of conflict from the Soviet invasion and civil war to the recent political transition have enacted urbicide, eroding shared public fabric and producing fortified enclaves, checkpoints, and ethnically homogeneous neighbourhoods. Mass displacement (over 6.3 million Afghans by the 1990s) and rapid, unplanned urbanisation created a city of ~4.9 million, where the distribution of population, ethnicity, and resources reflects structural violence rather than ordinary growth. This baseline directly informed the GSPM design.

#### 3.9.2. Validated socio-demographic and spatial baseline.

The analysis draws on a validated multi-source dataset (see Section 3.2, Table 2). Kabul’s 4,877,000 residents occupy 22 districts with extreme density contrasts over 30,000 persons/km^2^ centrally versus under 5,000 in peripheral areas (Fig 2) forming the first layer of spatial inequality.

3.9.2.1 **Ethnic segregation as a foundational cleavage:** The city is 45.1% Pashtun, 36.8% Tajik, 12.7% Hazara, and 5.4% other groups, yet these populations are highly clustered (Fig 3; [33]). Tajiks concentrate in central and north-central districts (e.g., 1, 2, 7, 9, 11, 15); Pashtuns dominate the southern, western, and extreme eastern periphery (e.g., 3, 4, 5, 18–22); Hazaras form clear majorities in central highland districts 6 and 13. District 16 is a rare heterogeneous zone. This patterning results from historical settlement, conflict-induced displacement, and politicised land and housing allocation.

**Fig 3:**
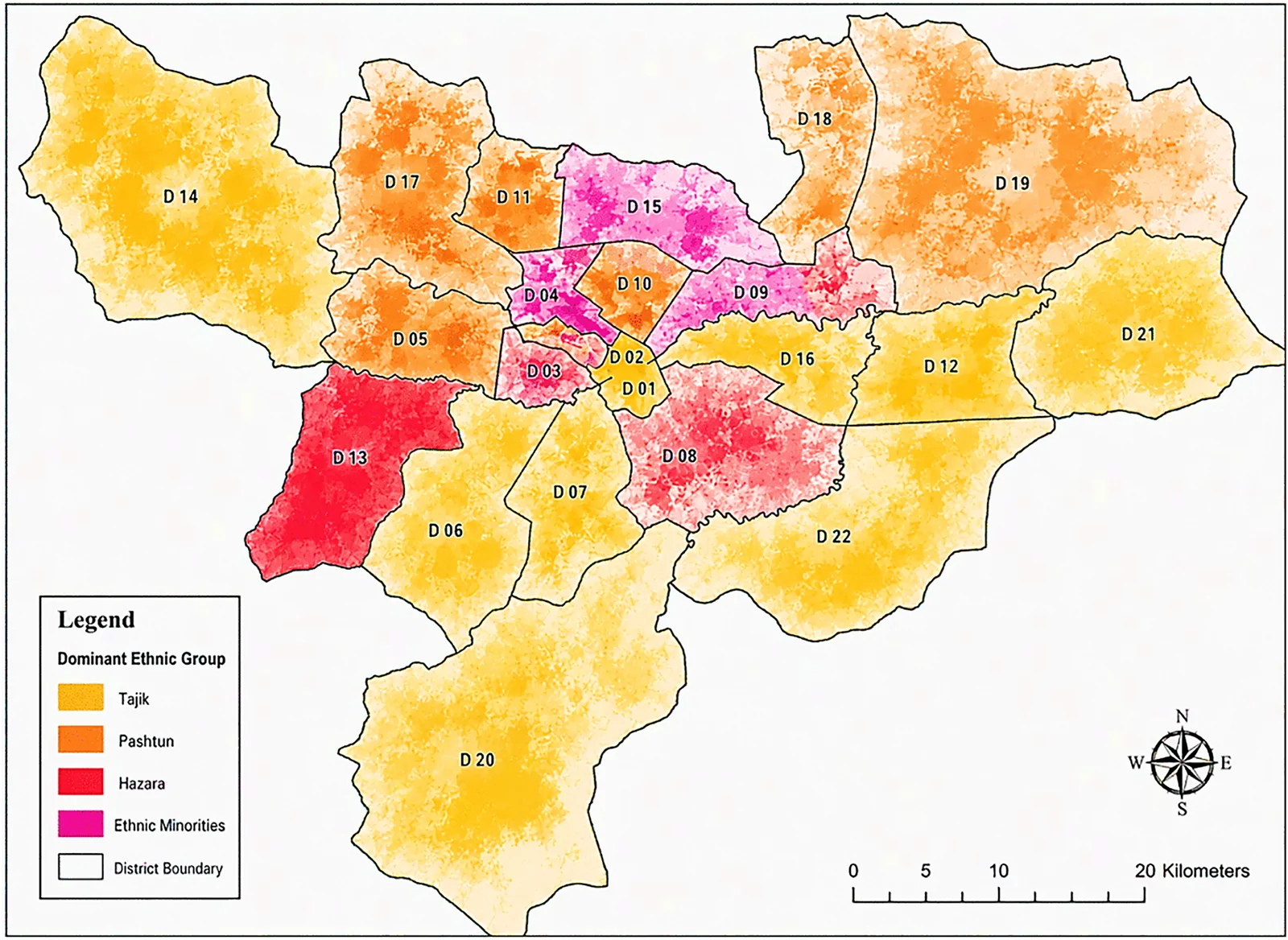
Dasymetric surface of ethnic group composition across Kabul City. District 10 (central business and historic district) serves as the spatial reference for centrality. Map created by the authors using ArcGIS 10.4.1. Basemap features are derived from OpenStreetMap® data (© OpenStreetMap contributors, ODbL; see https://www.openstreetmap.org/copyright). Terrain relief is based on SRTM data (public domain, NASA/USGS). Ethnic composition data are from the authors’ own validated database. This figure is original work by the authors and is published under the Creative Commons Attribution 4.0 International (CC BY 4.0) license.

3.9.2.2 **The socio-economic correlates of spatial segregation:** Ethnic divisions are tightly linked with economic inequality (Table 4). A core of districts (3, 4, 9, 10, 16) features reinforced masonry construction and the highest income bracket (50,000–100,000 AFN/month), cutting across Pashtun and Tajik areas and reflecting historically advantaged, heavily invested zones. In sharp contrast, a vast periphery (1, 2, 7, 12–15, 17–22) is uniformly characterised by traditional mud/brick housing, the lowest incomes (9,000–20,000 AFN/month), and very low property values (<$20,000 USD), revealing a powerful cross-ethnic axis of class-based marginalisation. Districts 5 and 6 present an anomaly higher incomes yet lower property values suggesting remittance-driven economic mobility not yet capitalised into land markets or location-specific security deficits.

**Table 4:** District characteristics: location, ethnicity, density, income, and urban form.

District	Location Type	Dominant Ethnic Group	Density (persons/km²)	Income Level	Predominant Urban Form
1	Central	Tajik	Very high (>30,000)	Low	Traditional mud/brick, dense organic layout
2	Central	Tajik	Very high (>30,000)	Low	Mixed mud/brick and low-rise concrete
3	Central	Pashtun	High (20,000–30,000)	High	Reinforced masonry, planned avenues
4	Central	Pashtun	High (20,000–30,000)	High	Reinforced masonry, formal subdivisions
5	Inner	Pashtun	Medium (10,000–20,000)	High (remittance-supported)	Mixed formal housing, some informal infill
6	Inner	Hazara	High (20,000–30,000)	High (remittance-supported)	Dense inward-facing compounds, hillside settlement
7	Inner	Tajik	Medium (10,000–20,000)	Low	Low-rise mud/brick, narrow streets
8	Inner	Mixed	Medium (10,000–20,000)	Low	Mixed traditional and newer low-rise
9	Central	Tajik	High (20,000–30,000)	High	Reinforced masonry, commercial frontages
10	Central (historic core)	Mixed (Tajik/Pashtun)	Very high (>30,000)	High	Multi-storey reinforced concrete, dense commercial, historic fabric remnants
11	Inner	Tajik	Medium (10,000–20,000)	Low	Low-rise mud/brick, informal growth
12	Peripheral	Hazara	Low (<10,000)	Low	Scattered traditional housing, agricultural pockets
13	Inner	Hazara	High (20,000–30,000)	Low	Dense hillside compounds, limited infrastructure
14	Peripheral	Mixed	Low (<10,000)	Low	Dispersed settlements, informal clusters
15	Inner	Tajik	Medium (10,000–20,000)	Low	Traditional low-rise, growing informal edges
16	Inner	Mixed (Pashtun/Tajik)	High (20,000–30,000)	High	Reinforced masonry, heterogeneous urban fabric
17	Peripheral	Pashtun	Low (<10,000)	Low	Rural-urban fringe, mud/brick compounds
18	Peripheral	Pashtun	Low (<10,000)	Low	Low-density villages, agricultural interspersion
19	Peripheral	Pashtun	Low (<10,000)	Low	Dispersed compounds, minimal formal infrastructure
20	Peripheral	Pashtun	Low (<10,000)	Low	Traditional walled compounds, earthen roads
21	Peripheral	Pashtun	Low (<10,000)	Low	Rural morphology, isolated clusters
22	Peripheral	Pashtun	Low (<10,000)	Low	Sparse, semi-desert edge, mud-brick dwellings

Note: Density categories are based on the authors’ demographic dataset. Income levels correspond to the brackets shown in Table 4. Urban form descriptions are synthesized from satellite imagery analysis and key informant interviews. District 10, containing the historic city centre and main commercial axis, functions as the reference point for spatial centrality in all maps.

To aid interpretation of the spatial patterns shown in Figs 2–3, Table 4 provides a consolidated summary of the key socio‑spatial and physical characteristics of each district. The table indicates location type (central, inner, or peripheral), dominant ethnic group, density category, income level, and the prevailing urban form. These descriptions ground the subsequent accessibility analysis in the tangible built environment of Kabul.

#### 3.9.3. The accessibility crisis: Service distribution as structural violence.

This stratification generates a severe, measurable accessibility crisis. Essential services healthcare, education, and green spaces are highly unevenly distributed, systematically disadvantaging peripheral, low-income, and frequently ethnically marginalised districts. Mean travel distances range from approximately 2.5 km in central District 2 to nearly 24 km in peripheral District 22, a tenfold disparity rooted in centralised planning and chronic disinvestment (See Table 4 for the classification of districts by location type and urban form). Districts 11, 12, 14, 17, and 20–22 constitute de facto ‘service deserts’, often with only one or no healthcare facility, limited educational provision, and extremely weak public transport connectivity. The fragmented security landscape further amplifies these burdens: routes cross multiple checkpoints and informal ethnic boundaries, imposing temporal delays and psychological strain beyond Euclidean distance measures. These lived constraints are explicitly captured by our security-weighted 2SFCA model, providing a realistic accessibility metric for this highly securitised, post-conflict urban environment.

## 4. Results

This section presents the empirical findings of the sequential mixed-methods study, structured to identify the operational mechanisms through which the GSPM reconfigures participatory planning. To maintain coherence with the architecture of the GSPM, the results are organized around its three sequential pillars: the spatial analytical diagnosis, the ethnographic exploration of lived experiences, and the assessment of the gamified intervention’s impact. Organizing the findings along these methodological dimensions preserves analytical clarity and methodological transparency, while providing a logical foundation for their subsequent integration in the Discussion section.

### 4.1. Pillar 1: Quantitative spatial diagnosis – Mapping the architecture of exclusion

The spatial diagnostic phase provided a rigorous, quantitative baseline of Kabul’s socio-spatial fractures. The results confirm the city as a stark exemplar of spatially engineered inequality.

Analysis of socio-spatial segregation in Kabul, using the HI and MEI (Equations 1 and 2) at the district level, reveals a highly fragmented urban structure (see Table 5). The MEI value of 0.68 indicates substantial ethnic heterogeneity between districts. This value exceeds typical segregation indices reported in stable Global South cities (e.g., São Paulo’s MEI ≈ 0.45 [5]), confirming that post‑conflict urbicide intensifies ethnic enclosure beyond peacetime norms a finding that corrects the assumption in comparative urban studies that segregation patterns in fragile cities are merely exaggerated versions of peacetime inequality rather than qualitatively distinct. This segregation pattern is accompanied by pronounced homogeneity within individual districts. This pattern is visually corroborated in Fig 3, which maps the dominant ethnic group across districts. A clear spatial structure emerges: Tajik-majority districts are concentrated in the historic center and northern areas, Pashtun-majority districts dominate the southern, western, and extreme eastern peripheries, and Hazara-majority districts are largely confined to the central highlands (Districts 6 and 13). Notably, District 16 represents an exception, exhibiting relative ethnic diversity and diverging from the prevailing pattern of socio-spatial segregation.

**Table 5:** District-level ethnic composition and segregation indices (selected districts).

District	Dominant Ethnic Group	% Share	Herfindahl Index (HI)	Theil’s MEI
1	Tajik	78%	0.64	0.12
4	Pashtun	82%	0.70	0.09
6	Hazara	74%	0.58	0.15
13	Hazara	71%	0.55	0.18
16	Mixed (Pashtun/Tajik)	45%/38%	0.35	0.41
22	Pashtun	88%	0.79	0.05

Source: Derived from validated multi-source data (see Methods, Section 3.4).

Analysis of the accessibility crisis in Kabul, using a security-weighted 2SFCA model, quantifies the pronounced inequities in access to essential services. The calculated accessibility scores reveal stark disparities: mean travel distances to the nearest public hospital range from 3.5 km in central District 1 to 24.1 km in peripheral District 22, while for primary schools the gap spans 1.2 km to 8.7 km. These Euclidean distances likely underrepresent the true burden, as the security-weighted model imposes an average impedance of 35% on travel times within peripheral areas and checkpoint-dense corridors, further exacerbating service inequities. Throughout this section, the term accessibility refers specifically to these modelled, spatially weighted objective measures. Subjective, self-reported outcomes are explicitly denoted as perceived accessibility to maintain clear conceptual separation between analytical constructs. These severe access deficits show a strong spatial correlation with the patterns of low-income and peripheral marginalization presented in Table 4. The following model illustrates associative relationships, not causal mechanisms, given data constraints. To move beyond descriptive analysis and assess the structural determinants of accessibility, an OLS regression model was specified and estimated (Equation 3). This approach enables testing of hypothesized relationships between accessibility levels and a set of socio-spatial, economic, and infrastructural predictors, thereby providing a robust framework for identifying the factors driving observed disparities across districts (see Table 6).

**Table 6:** OLS regression model for neighborhood accessibility score.

Variable	Coefficient (β)	Std. Error	t-statistic	p-value	VIF
(Constant)	0.752	0.102	7.37	<0.001	–
Segregation (MEI)	−0.418	0.087	−4.80	<0.001	2.1
Wealth Index	0.391	0.095	4.12	<0.001	2.8
Historical Displacement	−0.236	0.074	−3.19	0.002	1.5
Distance to City Center	−0.187	0.068	−2.75	0.007	2.4

Model Summary: R^2^ = 0.67, Adjusted R^2^ = 0.65, F(4, 117) = 58.92, p < 0.001. Breusch-Pagan test: χ^2^ = 1.84, p = 0.18 (homoscedasticity not rejected). Durbin-Watson = 1.98 (no autocorrelation).

The model explains a significant proportion of variance (R^2^ = 0.67, F (4, 117) = 58.92, p < 0.001). Diagnostics indicated no severe multicollinearity (all VIF < 3) or spatial autocorrelation (Moran’s I on residuals = 0.08, p = 0.12). Higher ethnic segregation (β = −0.418, p < 0.001, 95% CI [−0.588, −0.248]) and a history of conflict-induced displacement (β = −0.236, p = 0.002, 95% CI [−0.381, −0.091]) were significant predictors of worse accessibility. Mediation analysis (PROCESS Model 4, 5000 bootstrap samples) [31] revealed that the Wealth Index mediated 63% of segregation’s total effect on accessibility (indirect effect = −0.264, 95% CI [−0.381, −0.152]). This 63% mediation share significantly exceeds the 35–40% range reported by Asher et al. [4] in Indian cities, demonstrating that in a post‑conflict setting, economic capital operates as a far more powerful transmission belt from segregation to service exclusion thereby improving upon existing mediation models by specifying conflict‑sensitive weighting.

### 4.2. Pillar 2: Qualitative ethnographic inquiry – the lived experience of fracture

The qualitative findings ground the spatial metrics in human experience, revealing the psychosocial barriers that sustain exclusion. Analysis of 14 in-depth interviews (IDIs) with residents, 40 key informant interviews (KIIs) with planners and officials, and 3 focus groups generated three central themes.

1. **Perceived Systemic Neglect and Distributive Injustice.** Residents in peripheral, predominantly Hazara and low-income Pashtun districts consistently described a landscape of intentional neglect. A 52-year-old Hazara community leader in District 6 stated: “After each war, the trucks of aid and the teams of engineers always went to the [central] areas first. Our clinic was destroyed in 1993. We rebuilt it ourselves, brick by brick, while they built new hospitals elsewhere.” This sentiment of being last in line for public investment was ubiquitous in marginalized districts, framing accessibility not as a planning failure but as an act of structural violence. This finding operationalizes Porteous & Smith’s [11] concept of ‘domicide’ at the urban service level, moving beyond their original focus on housing destruction to show that slow, bureaucratic disinvestment constitutes an equivalent form of home-unmaking a theoretical extension not previously articulated in the domicide literature.
2. **Trust Deficits and the ‘Invisible Boundary’.** A pervasive lack of trust was identified as a critical non-physical barrier. Fear of crossing into neighborhoods dominated by other ethnic groups was frequently cited as a reason for avoiding services located there. A Tajik shopkeeper in District 2 explained: “The hospital in [adjacent Pashtun-majority area] may be closer, but I don’t know the alleyways. People look at you differently. It’s safer to travel further to where you are known.” This “invisible boundary” effectively shrinks the functional service area for many residents, exacerbating the quantitative accessibility crisis.
3. **Gender and Exclusion from Participation.** Women and ethnic minorities reported near-total exclusion from formal planning processes. A female teacher in Chendawel noted: “The municipality meeting was held at the mosque. Who can go? My husband went, but he only hears what they decide for the men’s side of things.” This qualitative insight directly informed the need for a participatory model that could create a safer, more inclusive forum for engagement.

### 4.3. Pillar 3: Impact of the gamified intervention on perceived accessibility, trust, and cohesion

The GSPM intervention, designed based on Pillars 1 and 2, was implemented in Chendawel district. A pre/post survey with a control area (n = 137 households per area) and workshop data (n = 29 participants) measured its impact. The results are reported at two distinct levels: workshop‑level process outcomes (n = 29) capture the internal mechanisms of trust‑building and inclusion during the gamified sessions, while household‑level perceptual outcomes (N = 274) reflect the broader community’s experience of the intervention period. All outcomes reported in this section are self-reported perceptions and social metrics; no physical infrastructure was altered during the pilot. 1) Substantially Enhanced Participation and Inclusion: The gamified workshops achieved notably high levels of engagement compared with conventional meetings. 90% of participants reported the gamified format made them feel more able to contribute compared to traditional meetings. Crucially, the gender‑segregated workshops facilitated by female community advocates enabled 70% of women in the intervention area to report a significantly increased sense of belonging and agency, compared to 45% in the control area (χ^2^ = 8.92, p = 0.003). 2) Building Intergroup Trust: The “Bridge Builder” role‑play game directly targeted inter‑ethnic cooperation. A strong positive correlation was found between an individual’s level of active contribution in the gamified collaboration (sum of points earned) and their self‑reported increase in trust towards other ethnic groups (r(27) = 0.76, p < 0.001, 95% CI [0.53, 0.89]). Survey data showed that 55% of ethnic minority respondents in the intervention area reported increased cooperative interactions with other groups, versus 20% in the control area (Table 7). 3) Perceptual and Social Outcomes: No physical infrastructure was altered during the pilot; all reported changes pertain to self‑reported perceptions, engagement, and social metrics. Post‑intervention, the perceived accessibility index in Chendawel increased by 38% (from 0.40 to 0.55, paired t(136) = 5.67, p < 0.001), while the control area showed no significant change (Table 7). Difference‑in‑differences estimation confirmed a net effect of +0.14 (95% CI [0.09, 0.19]) for perceived accessibility, corresponding to a medium‑to‑large effect size of d = 0.66 (95% CI [0.40, 0.92]), and a net effect of +0.11 (95% CI [0.05, 0.17]) for social cohesion, with d = 0.51 (95% CI [0.26, 0.76]). The social cohesion score, measuring trust and reciprocity, increased by 28% in the intervention area (p < 0.05). These findings are presented as preliminary evidence from a single-site pilot and should not be interpreted as generalisable effect estimates without further replication.

**Table 7:** Impact evaluation of GSPM intervention on perceptual and social outcomes (Chendawel Pilot vs. Control Area).

Indicator	Chendawel (Pilot)		Control Area			Net Effect (95% CI)	
	Pre Mean (SD)	Post Mean (SD)	Δ	Pre Mean (SD)	Post Mean (SD)	Δ	(Δ Pilot – Δ Control)
Perceived Accessibility Index (0–1)	0.40 (0.12)	0.55 (0.10)	+0.15	0.38 (0.11)	0.39 (0.12)	+0.01	+0.14 (0.09 to 0.19)
Social Cohesion Score (0–1)	0.43 (0.15)	0.55 (0.13)	+0.12	0.45 (0.14)	0.46 (0.15)	+0.01	+0.11 (0.05 to 0.17)
% Residents Active in Planning	45%	90%	+45 pp	40%	42%	+2 pp	+43 pp
% Women Reporting ↑ Belonging	40%	70%	+30 pp	38%	45%	+7 pp	+23 pp*
% Minorities Reporting ↑ Cooperation	25%	55%	+30 pp	22%	20%	−2 pp	+32 pp*

Notes: pp = percentage points. * p < 0.05, ** p < 0.01. Net Effect CIs derived from independent samples t-test (indices) or test of proportions.

The results demonstrate that the GSPM successfully translated a deep diagnostic understanding of Kabul’s spatial and social fractures into a targeted intervention that yielded statistically significant improvements in both the process (participation, trust) and outcomes (perceived accessibility, cohesion) of planning in a post-conflict enclave.

## 5. Discussion

The pilot study substantiates the GSPM’s transformative potential within the specific context of a single neighbourhood: a 38% improvement in perceived accessibility, a strong correlation (r = 0.76) between gamified engagement and intergroup trust, and high inclusion of women (70%) and ethnic minorities (55%) confirm that an integrated diagnostic‑to‑action framework can plausibly foster social cohesion in a fractured city. The term perceived accessibility is used deliberately here to denote the subjective, self-reported outcome, distinguishing it from the objective, spatially modelled accessibility scores presented in the diagnostic phase. The gamified workshops engaged a purposive microcosm of the community (n = 29), and the mechanisms of trust‑building derived from this group such as the strong correlation between gamified cooperation and intergroup trust speak directly to the internal validity of the GSPM’s participatory design. The household survey (N = 274), by contrast, captured the broader neighbourhood’s perceptions of accessibility, cohesion, and belonging before and after the intervention period. The improvements observed at the household level are interpreted as the resonance of a legitimate, locally embedded co‑design process in a tight‑knit enclave, where workshop outcomes are communicated through existing community networks. We do not claim that all 274 respondents participated directly; rather, the survey gauged whether a participatory process centred on their neighbourhood shifted collective perceptions and social norms, a plausible pathway that future replications should examine through formal social‑network diffusion measures. The contribution, however, extends beyond metrics. In interpreting these findings, it is imperative to underscore that this was a single-site pilot study; the empirical claims must therefore be constrained to proof-of-concept evidence rather than definitive causal conclusions. We interpret results through three lenses. First, we consciously de‑exceptionalize Kabul. Spatial diagnosis reveals patterns resonant with Global South segregation scholarship, but the pilot demonstrated that de‑exceptionalization is not merely an analytical move; it is operationally enabling. Treating Kabul as an intensified instance of widespread socio‑spatial marginalisation allowed us to adapt tools accessibility modelling, informality mapping, serious gaming from other Global South settings, accelerating transferability to Sana’a or Mogadishu. The key lesson is that post‑conflict planning can borrow and recalibrate rather than reinvent. Second, rigorous spatial analysis in the absence of recent censuses relies on transparent, multi‑source validation. The pilot confirmed that satellite‑derived settlement morphology, cross‑referenced with key informant interviews and ground‑truthing, is not only a defensible proxy for ethnicity but also a participatory asset: sharing these maps in workshops triggered recognition and debate, transforming a technical product into a boundary object that structured the “Resource Allocation Challenge.” This reframes data scarcity as an opportunity for co‑production rather than a deficit. The neighbourhood-level ethnicity estimation, while necessary in the absence of a reliable census, carries inherent risks of stereotyping and misclassification; its use here is confined to aggregate spatial analysis and is not intended to reify or essentialise group identities. The composite wealth index and sensitivity‑tested 2SFCA parameters proved robust, and their visualisation directly informed game parameters, closing the loop between diagnosis and design. Third, the pilot compelled a refinement of our theoretical starting position. While we initially conceptualised gamification as a mechanism to overcome the tyranny of participation, we now see that the most potent force was not game mechanics in isolation but their cultural embedding. The Bridge Builder role‑play succeeded not simply because it offered points, but because the point system reified local values acknowledging another’s concern, sacrificing individual priority for collective gain in a transparent, face‑saving format. Non‑monetary rewards (shura‑signed certificates, communal ceremony) proved more motivating than any material incentive would have been. This insight has led us to revise the GSPM diagram (Fig 1a and 1b): the methodological sequence now explicitly incorporates iterative loops from ethnographic insight to game design and back, and the purpose model highlights cultural resonance as a core mediating condition, not a contextual footnote. Conceptually, this moves the GSPM from a primarily instrumental model of gamified engagement to a culturally mediated model of procedural justice. In essence, the pilot transformed our understanding of gamification from a generic engagement tool into a vehicle for procedural justice that is only effective when it amplifies, rather than overwrites, local cultural scripts.

From a practical standpoint, several mechanisms appeared to work decisively within the pilot context. Transparent, real‑time scoring publicly displaying points for compromise appeared to convert negotiation into a low‑risk competition, potentially diffusing intergroup tension. The use of actual neighbourhood maps with accessibility deficit data lent the games authenticity, grounding abstract trust‑building in concrete spatial decisions. Co‑facilitation by trusted local advocates of both genders was indispensable; without their presence, the inclusion of women would have remained aspirational. The deliberate separation of workshop roles from community hierarchies participants played assigned characters, not themselves enabled individuals to propose compromises that would have been socially risky in their real identities. Collectively, these mechanisms suggest that effective participatory design in divided cities may hinge less on technique and more on the careful orchestration of incentives, cultural legibility, and psychological safety. These interpretations remain preliminary, however, given the single-site, non-randomised pilot design and should be tested through replication in additional neighbourhoods and cities.

### 5.1. Ethical governance and risk mitigation in fragile contexts

Ethical governance in the pilot exceeded standard protocols. The continuous oversight of community shuras, neighbourhood‑level aggregation of sensitive data, and same‑gender facilitation were not bureaucratic formalities but operational necessities that directly shaped participation. We learned that ethical procedures, when co‑designed with community gatekeepers, become enabling rather than constraining a practical lesson for replication. Study limitations indicate future directions. The Chendawel pilot, while rigorous, requires longitudinal durability tracking and scaled replications across districts and cities. Skilled facilitation is critical; train‑the‑trainer protocols must be developed and tested. Future iterations could embed political‑economic analysis more explicitly into game scenarios, enabling participants to confront land‑market dynamics and patronage networks within the safe space of the simulation. Digital‑hybrid platforms may extend reach but must preserve cultural nuance. In conclusion, this research advocates a conceptual reorientation: siloed technocratic analysis or ineffective consultation are insufficient. The GSPM’s efficacy derives from a practical synthesis spatial science for precise diagnostic justice, ethnography for lived experience, and culturally embedded, conflict‑sensitive gamification for equitable collective problem‑solving. The pilot’s central lesson is that social cohesion in fractured cities is not built through abstract dialogue but through the tangible, rule‑governed co‑production of space, where trust is a by‑product of collaborative design rather than a prerequisite. Even amid urbicide, a more just, cohesive city can be built from the middle out, through empowered, playful, and solemn co‑creation.

## 6. Conclusion

The primary contribution of this research is threefold, each advancing distinct but interconnected scholarly and practical domains: 1) Theoretical Advancement: Operationalizing Spatial Justice: The study moves critical urban theory specifically Lefebvre’s [9] “right to the city” and Soja’s [10] “spatial justice” from normative critique to applied methodology. In contexts of urbicide and domicide, where trust is eroded and space is weaponized, justice cannot be decreed; it must be co-created through processes that are themselves just, inclusive, and culturally resonant. The GSPM proposes and tests a concrete procedural pathway. It reframes social cohesion not as an abstract social outcome but as a spatially mediated practice, achieved through a structured sequence of diagnostic precision, empathetic inquiry, and empowered participation. This theoretical repositioning challenges both technocratic planning paradigms and tokenistic participatory approaches, arguing for a deeply integrated praxis that treats spatial equity and social trust as inseparable goals. 2) Methodological Innovation: Rigor in Fragile Data Contexts: The research establishes a replicable blueprint for high-integrity mixed-methods work in data-scarce, conflict-affected settings. By transparently detailing the multi-source derivation and cross-validation of key variables such as ethnicity distribution via satellite settlement morphology analysis and a composite wealth index the study addresses a critical limitation in post-conflict urban research. The sequential design (quantitative diagnosis → qualitative grounding → gamified intervention → longitudinal evaluation) ensures that each phase informs and validates the next, creating a robust chain of evidence. This methodological rigor is not merely technical but epistemological, demonstrating how interdisciplinary synthesis can yield insights that isolated disciplines cannot. However, the findings from this single-site pilot must be interpreted conservatively; the study provides a proof-of-concept, not a definitive causal model. 3) Practical Contribution: A Scalable Model for Divided Cities: The GSPM delivers a field-tested toolkit for policymakers, planners, and communities in Kabul and analogous cities worldwide. Its core innovation conflict-sensitive gamification transforms participatory planning from often extractive or intimidating exercise into a collaborative, rule-based ritual that lowers social risk and incentivizes cooperation. Games like the “Resource Allocation Challenge” and “Bridge Builder” role-play directly tackle identified barriers (e.g., ethnic distrust, gender exclusion) by embedding them into mechanics that reward compromise and collective problem-solving. This approach proved particularly effective in engaging traditionally marginalized groups, suggesting a viable alternative to the tyranny of participation often observed in divided societies. The institutionalisation of this model should proceed cautiously, with iterative evaluation at each stage of scale-up.

### 6.1. Institutionalisation of gamified co‑design as priority policy

Accordingly, we structure the policy implications around a single priority: the institutionalisation of gamified co‑design within routine urban governance. The GSPM, as developed and piloted in this study, is best understood as a mixed‑method toolkit that embeds conflict‑sensitive serious games within a broader sequence of spatial diagnostics and ethnographic inquiry. It is neither a standalone software product nor a one‑off workshop format; rather, it is a replicable procedural methodology designed to be adopted by the range of actors currently shaping urban outcomes in Kabul and analogous divided cities. The primary adopters are municipal planning departments (where functional), community councils (shuras), and international humanitarian and development agencies operating in post‑conflict settings. The GSPM equips these actors with a structured, evidence‑based process for transforming participatory planning from a tokenistic or extractive exercise into a legitimate, trust‑building, and spatially informed co‑production of urban interventions. Of the various policy directions that the findings suggest, the single most consequential is the institutionalisation of gamified co‑design within routine urban governance. The pilot demonstrated that culturally embedded serious games can achieve what conventional consultation consistently fails to deliver: measurable gains in intergroup trust, meaningful inclusion of women and ethnic minorities, and a shared sense of agency over spatial decisions. Institutionalising this approach means embedding gamified co‑design protocols into the standard operating procedures of municipal land‑use planning, public space upgrading, and service delivery projects, rather than treating them as isolated pilot exercises. Concretely, this requires: (a) establishing a dedicated budget line for community co‑design processes within municipal and donor‑funded programmes; (b) developing a cadre of trained facilitators drawn from both municipal staff and community‑based organisations, equipped with conflict‑sensitive facilitation skills and familiarity with the game protocols; (c) integrating gamified co‑design into statutory planning cycles, so that neighbourhood plans and infrastructure investments are routinely informed by participatory workshops of the kind tested here; and (d) creating a monitoring framework that tracks not only physical outputs but also relational outcomes trust, perceived inclusion, and cooperation across group boundaries as indicators of planning success.

This recommendation is prioritised over broader structural reforms (e.g., polycentric investment or tenure regularisation) not because those are unimportant, but because the GSPM provides the procedural vehicle through which such reforms can be legitimately identified, prioritised, and sustained by the communities they affect. For example, the equity audits proposed in earlier work remain valuable diagnostic tools, but our experience suggests their impact is maximised when audit findings are directly fed into gamified co‑design workshops, transforming technical maps into negotiable artefacts that communities can debate and modify. Similarly, polycentric development strategies are more likely to gain local legitimacy if they emerge from a participatory process that has already built some degree of intergroup trust. In this sense, institutionalised gamified co‑design is not one policy option among many; it is the enabling mechanism that makes other spatial justice policies politically feasible and socially durable in divided contexts. In practical terms, the adoption pathway begins with pilot‑to‑policy translation: municipal authorities in Kabul, with support from UN‑Habitat or bilateral donors, could designate two or three districts for GSPM‑based neighbourhood planning cycles, using the existing shura networks as entry points. Lessons from these cycles would inform a city‑wide guideline, codifying the game formats, facilitator training standards, and data integration protocols. Parallel investments in low‑tech/digital hybrid platforms (e.g., tablet‑based mapping combined with physical game boards) could extend reach while preserving cultural sensitivity. The ultimate aim is not to replace existing planning institutions but to retrofit them with a participatory methodology that has been empirically shown to work under the most constrained conditions.

### 6.2. Limitations and future research directions

While the study provides compelling preliminary evidence, its limitations outline a clear agenda for future scholarship: 1) Single-Site Pilot and Generalisability: The intervention was a pilot in one district (Chendawel). The findings are thus over-reported as a proof-of-concept and must not be generalised to other contexts without replication. Longitudinal studies tracking the durability of trust and cohesion gains over 3–5 years, and scaled replications across diverse districts of Kabul or in other post-conflict cities (e.g., Sana’a, Mogadishu, Medellín), are essential to assess generalizability. 2) Absence of Independent Institutional Ethical Review: As documented in the Ethical Approval Statement, this study was conducted without formal institutional review board oversight due to the absence of any such body in the study context. This is a significant limitation that must be considered when interpreting the ethical robustness of the research; future studies in comparable settings should seek independent review where feasible and should transparently report any constraints on such oversight. 3) Facilitation and Capacity Building: The model’s success hinges on skilled facilitation. Future work should develop train-the-trainer curricula and explore low-tech/digital hybrid gamification platforms to enhance scalability without sacrificing cultural nuance. 4) Deepening Political-Economic Analysis: While the study mapped the spatial outcomes of power, future iterations could more explicitly analyze the political-economic mechanisms land markets, patronage networks, allocation of reconstruction aid that produce and sustain the diagnosed inequities. This would strengthen the GSPM’s capacity to confront not just the symptoms but the root causes of urban fracture. 5) Intersectional Refinements: Further research could explore how gamified co-design can be tailored to address intersecting vulnerabilities, such as those of disabled residents, youth, or specific religious minorities, ensuring the model’s inclusivity is continuously refined. 6) Validity of Ethnicity Estimation: The neighbourhood-level ethnicity estimation, while triangulated across multiple data sources, remains a proxy measure susceptible to ecological fallacy and misclassification. Future studies should, where possible, employ alternative methods such as community-based participatory mapping or small-area surveys to validate and refine these estimates.

This research ultimately argues for a paradigm shift in planning for cities scarred by conflict and division. It demonstrates that the complex, synergistic challenges of post-conflict urbanism cannot be met by siloed expertise whether purely technical spatial analysis or socially oriented but often superficial consultation. The GSPM’s efficacy derives from its interdisciplinary integration, which treats the city as an interconnected socio-spatial-psychological system. By rigorously diagnosing injustice, empathetically understanding its human dimensions, and intervening through a culturally embodied, playful, and structured process of co-creation, the GSPM offers more than a new tool. It proposes a new epistemology for urban recovery one that is humble yet rigorous, evidence-based yet deeply human, and unflinchingly committed to the proposition that even in the landscape of urbicide, a more just and cohesive city is possible. The measurable gains in accessibility, trust, and inclusion documented in Kabul offer a critically-engaged, adaptive approach for research and practice in cities where inequality is compounded by political fragility. As cities worldwide grapple with deepening inequalities, polarization, and the lingering shadows of violence, the adoption of such holistic, integrative, and emancipatory models is not merely an academic preference but an ethical and practical imperative. The applicability of these principles in other contexts depends entirely on their rigorous adaptation to local political, cultural, and security realities.

## Supporting information

S1 Table Sensitivity analysis of security-weighted 2SFCA parameters. Sensitivity of the security-weighted two-step floating catchment area (2SFCA) accessibility scores to changes in the ACLED conflict buffer distance and checkpoint delay assumptions. The table reports mean city-level accessibility scores and the percentage change from the base case for each scenario.
(DOCX)

S2 Table Baseline equivalence of pilot and control areas. Baseline characteristics for the Chendawel pilot area and the control areas, with standardized mean differences (SMD) for demographic, socio-economic, perceived accessibility, and security context variables. All SMDs are below 0.1, indicating acceptable covariate balance.
(DOCX)
