# Supplementary material for: Gamified spatial‑participatory model for urban social cohesion in a post‑conflict city: Bridging accessibility, inclusion, and intergroup trust through co‑design in Kabul
Source: PLoS One. 2026 Oct 27;21(10):e0358692. doi: 10.1371/journal.pone.0358692 (PMC13712617; doi:10.1371/journal.pone.0358692)
Supplement: Supplementary file 1 [file pone.0358692.s001.docx]

**Table S1. Sensitivity Analysis of Security-Weighted 2SFCA Parameters.**

| Scenario | ACLED Buffer | Checkpoint Delay | Mean Accessibility Score (City) | % Change from Base |
| --- | --- | --- | --- | --- |
| Base Case | 1.0 km | +15 minutes | 0.42 | - |
| Sensitivity 1 | 0.5 km | +15 minutes | 0.43 | +2.4% |
| Sensitivity 2 | 1.5 km | +15 minutes | 0.41 | -2.4% |
| Sensitivity 3 | 1.0 km | +10 minutes | 0.43 | +2.4% |
| Sensitivity 4 | 1.0 km | +20 minutes | 0.40 | -4.8% |
