# Supplementary material for: Gamified spatial‑participatory model for urban social cohesion in a post‑conflict city: Bridging accessibility, inclusion, and intergroup trust through co‑design in Kabul
Source: PLoS One. 2026 Oct 27;21(10):e0358692. doi: 10.1371/journal.pone.0358692 (PMC13712617; doi:10.1371/journal.pone.0358692)
Supplement: Supplementary file 2 [file pone.0358692.s002.docx]

**Table S2. Baseline Equivalence of Pilot and Control Areas.**

| Variable | Chendawel (Pilot) Mean (SD) | Control Area Mean (SD) | Standardized Mean Difference (SMD) |
| --- | --- | --- | --- |
| Demographic |  |  |  |
| % Households Hazara | 92.5% (4.2) | 90.1% (4.8) | 0.053 |
| Mean Household Size | 7.1 (1.8) | 6.9 (1.7) | 0.058 |
| Socio-Economic |  |  |  |
| Composite Wealth Index (0-1) | 0.33 (0.11) | 0.31 (0.10) | 0.071 |
| Baseline Perceived Accessibility |  |  |  |
| Perceived Accessibility Score (0-1) | 0.40 (0.12) | 0.38 (0.11) | 0.075 |
| Security Context |  |  |  |
| # Checkpoints within 1km | 2.7 (0.6) | 2.6 (0.7) | 0.064 |
